# Exiting the pandemic: Singapore style

**DOI:** 10.1186/s12916-021-02117-y

**Published:** 2021-09-17

**Authors:** Dale Fisher, Kenneth Mak

**Affiliations:** 1 Infectious Diseases Division, National University Hospital, Singapore, Singapore; 2 Yong Loo Lin School of Medicine, National University of Singapore, Level 10, NUHS, 1E Kent Ridge Rd, Singapore, 119228 Singapore; 3 Ministry of Health, Singapore, Singapore

**Keywords:** COVID, Singapore, Exit, Strategy

## Background

Countries fortunate enough to have high vaccination rates are displaying varying pandemic exit strategies. The debate revolves around two factors. There are arbitrary vaccination rates targeted by countries despite the knowledge that herd immunity cannot exist with these vaccines due to their limited capacity to prevent transmission. Additionally, the role of non-pharmaceutical indications, including social restrictions that are required to enable a sustainable near normal “business as usual”, is questioned, in the post-vaccination era.

## Preparedness, readiness and a response balancing public health measures

More than 80% of Singapore’s total population has now received two doses of vaccination, a far cry from its situation in April 2020 when faced with an explosive situation in the foreign worker dormitories. In the subsequent 5 months, over 50,000 cases were diagnosed. A series of strategies led to a remarkably low death rate, discordant with the high case numbers [1]. The residents of the dormitories were isolated from the main community which separately recorded maximum daily case numbers of just 51 on April 8, the highest community level in 2020, just after the introduction of what would become the nation’s most extreme social restrictions of the pandemic [2].

It could be said that Singapore’s response began in 2003 in the wake of its SARS outbreak. Significant reforms to its Infectious Diseases Act [3] were made to support expanded detection and surveillance of communicable diseases, contact tracing and epidemiological investigations, as well as quarantine and isolation of cases to disrupt community transmission. Plans were put in place to renovate existing hospitals and build new facilities (including the National Centre for Infectious Diseases) consistent with pandemic principles of infrastructure expandability, convertibility and adaptability to cope with a major infectious disease outbreak [4]. Other features in the preparedness phase included human resources’ recruitment and internal development, regular pandemic exercises and stockpiling of drugs and PPE [5].

The readiness phase began when the Ministry of Health distributed its first guidance on January 2, 2020 [6]. On January 23, 2020, as the nation’s first case was identified in a traveller from Wuhan, a multi-ministry taskforce first met to set a tone of a whole of government response [7]. By this stage, Singapore’s hospitals had spent 3 weeks reviewing and adapting its processes in patient care, notably its infection prevention and control systems. Testing was available in many sites. Surge capacities were in place [8].

The response phase has relied significantly on border controls and social restrictions supporting a strong public health system whereby all cases have been isolated or cohorted in hospital or community facilities (including designated hotels). Contact tracing has never been overwhelmed and now is supported by efficient mobile phone-based tracking and check in systems. Contacts are usually accommodated in government quarantine facilities (including designated hotels) or can be permitted at home (with strong legal deterrents preventing breaches).

The community strategy in Singapore has been to minimise transmission via mandated mask wearing and restrictions on attendee numbers at large gatherings and mask-off activities such as dining, bars and gyms. Businesses and individuals have been financially supported. Work from home has been the default.

As a result of these strategies, breaches at border interfaces have not translated to uncontrolled spread. While Singapore has had many “zero case” days, there have been many isolated cases and small clusters which did not amplify [9]. An introduction of the delta variant recently has given rise to many clusters with daily numbers now increasing as restrictions ease. With this framework, Singapore has not had to reimpose the restrictions of April 2020, and no hospital has been overwhelmed.

The vaccination programme began on December 30, 2020. Dialog had existed between Singapore and several vaccine developers including Pfizer-BioNTech and Moderna throughout 2020 and was well placed to secure reliable vaccine supplies. Strategic distribution and messaging have seen strong uptake such that by August 29, the total rate of completion of the full vaccination regime amongst the entire resident population was 80% and another 3% pending a second dose.

Singapore has now begun a strategic graduated exit from the pandemic amongst a strong sense of community optimism due to very high vaccination rates and strong public health systems. Policies factoring in vaccine status will determine the size of gatherings at events such as weddings, concerts and religious gatherings during this transition. Differentiation within policies requires the unvaccinated to have a pre-event test or alternatively be limited to smaller group activities. Travel restrictions are easing with nuanced testing and quarantine requirements, and a vaccinated travel lane has been established to selected countries, where quarantine is waived. Vaccination is unlikely to be mandated, but the requirement for extra (unfunded) testing for the unvaccinated, before activities deemed at higher risk for exposure and transmission, will provide additional incentive to this small number of individuals. Singapore, the government and the population as one have a belief in the decision-making and messaging processes. A stepwise graduated and cautious exit strategy has been articulated not in detail but as a principle through an understanding that a reckless removal of all restrictions is to risk a (so far) lauded outcome in terms of health, social and economic balance.

## Conclusion

There is great trust in government that has navigated the pandemic with just 55 deaths by August 30, 2021, in this second densest country in the world of 5.7 million residents. Deliberate multifaceted risk communications and community engagement has featured throughout the pandemic and should enable patience from the population through this next phase. Singapore’s approach will involve several months of stepwise calibrated easing so that as restrictions are lifted and community case numbers increase, the impact on the healthcare system can be monitored and mitigated.

In Singapore, you will not see ‘Freedom Day’ rather, we will walk stone by stone, as we carefully cross the river [10].

## Data Availability

Not applicable
